# A Comparative Study on the Efficacy of Solifenacin Succinate in Patients with Urinary Frequency with or without Urgency

**DOI:** 10.1371/journal.pone.0112063

**Published:** 2014-11-17

**Authors:** Ji-Yeon Han, Kyu-Sung Lee, Won Hee Park, Choal Hee Park, Jeong Gu Lee, Jeong Zoo Lee, Duk Yoon Kim, Yong Gil Na, Dong Deuk Kwon, Myung-Soo Choo

**Affiliations:** 1 Department of Urology, Pusan National University Yangsan Hospital, Yangsan, Korea; 2 Department of Urology, Samsung Medical Center, Sungkyunkwan University School of Medicine, Seoul, Korea; 3 Department of Urology, Inha University College of Medicine, Incheon, Korea; 4 Department of Urology, Keimyung University School of Medicine, Daegu, Korea; 5 Department of Urology, Korea University College of Medicine, Seoul, Korea; 6 Department of Urology, Pusan National University Hospital, Busan, Korea; 7 Department of Urology, Catholic University of Daegu School of Medicine, Daegu, Korea; 8 Department of Urology, Chungnam National University School of Medicine, Daejeon, Korea; 9 Department of Urology, Chonnam National University Hwasun Hospital, Chonnam National University Medical School, Gwangju, Korea; 10 Department of Urology, University of Ulsan College of Medicine, Asan Medical Center, Seoul, Korea; Weill Cornell Medical College Qatar, Qatar

## Abstract

**Objectives:**

Patients with overactive bladder (OAB) often have trouble perceiving urgency because of difficulties in distinguishing between urgency and desire to void. Empirical antimuscarinic treatment of patients with frequency only may be reasonable if conservative management has failed. We compared the efficacy of solifenacin in patients with frequency with or without urgency.

**Materials and Methods:**

This multicenter, 12-week, open-label, comparative, non-inferiority clinical trial assessed whether the solifenacin efficacy for frequency without urgency is non-inferior to its efficacy for frequency with urgency. All patients had micturition frequency ≥8 voids/day with or without urgency. Primary efficacy variable: daily frequency change at 12 weeks relative to baseline. Secondary efficacy variables: change at 12 weeks relative to baseline in Patients' Perception of Bladder Condition (PPBC), OAB Symptom Score (OABSS), and Benefit, Satisfaction, Willingness to continue (BSW) questionnaire.

**Results:**

Of the 286 enrolled patients, 240 (83.9%) completed the study (without urgency n = 115; with urgency n = 125). Full dataset analysis revealed that the groups without and with urgency exhibited significant reductions in daily micturition frequency of −2.49±0.35 (mean ± standard error) and −2.63±0.37, respectively. The lower limit of the 95% two-sided CI of the comparison of the two group means was −1.14, which is smaller than the −0.8 margin of clinical equivalence. The two groups did not differ in improvement in PPBC, OABSS, or BSW scores. Both tolerated the treatment well.

**Conclusions:**

It was not possible to verify that the solifenacin efficacy for frequency alone was non-inferior to its efficacy for OAB. Nevertheless, solifenacin tended to be effective for frequency regardless of urgency.

**Trial Registration:**

ClinicalTrials.gov NCT00979472

## Introduction

Overactive bladder (OAB) is defined by the International Continence Society (ICS) as urgency [with or without urgency urinary incontinence (UUI)] that is usually associated with frequency and nocturia [1]. This definition suggests that urgency is the key symptom for a diagnosis of OAB, and as such, OAB cannot be diagnosed in the absence of urgency and is thought to be a driver for all other symptoms of OAB including frequency, nocturia, and UUI [2]. Due to its primary role in defining the OAB syndrome, it is important for clinicians to have a reasonable understanding of the definition of urgency.

The ICS defines urgency as “the complaint of a sudden compelling desire to pass urine which is difficult to defer” [1]. This assumes that urgency is an abnormal or pathological bladder sensation that is distinguishable from the normal physiological sensation of urge to void [2], [3]. In most studies of OAB, diagnosis was based on physician identification of urgency according to the ICS definition, and urgency was the primary endpoint. However, greater clarity has been needed in the development of instruments for measuring urgency, because it is generally difficult for patient with OAB to perceive urgency and to differentiate urgency from urge to void [4].

In clinical experience, it seems that many patients who present with urinary frequency complain of a desire to void without urgency as defined by ICS, yet it is not suited for the current ICS definition of OAB. Nevertheless, despite the fact that urgency is the key symptom in OAB, frequency is also regarded as one of the most bothersome OAB symptom [5]. Despite this, the pathophysiology of frequency only and treatment guidelines for the management of patients with frequency only have not been established.

We hypothesized that the some of the patients who complained of only frequency without urgency may be actually OAB patient who cannot perceive or express their urgency symptoms, and antimuscarinic drugs may be effective in patients who complained of only urinary frequency without urgency, as they are in patients with urgency. Thus, we compared the efficacy of solifenacin in patients with frequency only and frequency with urgency.

## Materials and Methods

The protocol for this trial and supporting CONSORT checklist are available as supporting information; see Checklist S1, Protocol S1 and Protocol S2.

### 2.1. Study design

This multicenter, 12-week, open-label, comparative, non-inferiority study was based on the hypothesis, “The efficacy of solifenacin for frequency only is non-inferior to the efficacy of solifenacin for frequency with urgency”. It was conducted at nine sites between April 2009 and September 2011 in Korea. The study was conducted in accordance with the ethical principles in Korean Good Clinical Practice, the Declaration of Helsinki, and the International Conference of Harmonization Guidelines. This study was approved by the Institutional Review Board of Asan Medical Center (No. 2009-0014). Before enrollment in the study, all patients provided written informed consent.

### 2.2. Study patients

The study population consisted of men and women aged ≥18 years with symptoms of frequency for more than 3 months. All patients were assessed by a 3-day voiding diary and the urinary sensation scale (USS) [6]. The voiding diary and USS were carefully instructed by a study nurse who had been trained and explained the meaning of urgency and USS to all patients. Recording in voiding diary included day and night frequency, voided volume, and USS for each void. In the USS, the grade of urinary sensation perception was defined by scores from 1 to 5 as follows: 1 =  no urgency: no feeling of urgency (can continue activities until it is convenient to use bathroom); 2 =  mild urgency: feel urgency (can easily tolerate; can finish usual activity and tasks quickly, and then go straight to the bathroom); 3 =  moderate urgency: enough urgency discomfort (need to stop usual activity and tasks, and go straight to the bathroom); 4 =  severe urgency: strong urgency discomfort (almost cannot hold urine; need to stop usual activity and tasks immediately, and run to bathroom to avoid a micturition accident); and 5 =  urge incontinence: extreme urgency discomfort (cannot hold urine, and has a micturition accident before reaching the bathroom). The mean score of USS recorded on the 3-day voiding diary was considered as the USS for the patient, and urgency was defined as USS ≥3 based on a voiding diary.

The Patients were excluded if they had: significant stress urinary incontinence, an average total daily urine volume >3000 ml, serum liver enzymes or creatinine level >2 times the upper limit of normal, symptomatic urinary tract infection at screening, recurrent urinary tract infections (defined as receiving treatment for symptomatic urinary tract infections >4 times in the last year), interstitial cystitis, urothelial tumor, a post-void residual (PVR) urine volume >100 ml, clinically relevant bladder outlet obstruction, clinically significant pelvic organ prolapse, electrostimulation treatment, undergone bladder training in the preceding 2 weeks, received antimuscarinic medication in the preceding 2 weeks, and/or neurological conditions that can specifically affect bladder function.

### 2.3. Interventions

We classified the patients with average micturition frequency ≥8/24 hours without urgency as Group 1, and patients with average micturition frequency ≥8/24 hours with urgency (USS ≥3/3 days) as Group 2, based on a 3-day voiding diary. Patients received solifenacin 5 mg once daily. At the week 4 visit, the dose could be increased to 10 mg based on discussion between the subject and investigator regarding treatment efficacy and tolerability.

### 2.4. Efficacy and safety assessments

To assess efficacy, the patients completed a 3-day voiding diary before the clinic visits at baseline and at weeks 4 and 12 (final visit). The primary efficacy variable was change in daily micturition frequency at 12 weeks relative to baseline. The secondary efficacy variables were change from baseline in Patients' Perception of Bladder Condition (PPBC) [7], OAB Symptom Score (OABSS) [8], and Benefit, Satisfaction, and Willingness to continue (BSW) scores, as determined by questionnaires completed at baseline and the end of treatment. Safety was evaluated by recording adverse events and measuring maximal urinary flow rate (MFR) and PVR.

### 2.5. Statistical analyses

Sample size was calculated by using the Power Analysis and Sample Size (PASS) statistical software package (PASS 11, NCSS, LLC, Kaysville, UT, USA). Sample sizes of 128 evaluable patients per treatment group were deemed to provide approximately 80% power to detect a non-inferiority margin of equivalence of −0.8 (standard deviation: 2.56 [9]) in terms of change in mean micturitions per 24 hours when Group 1 was compared to Group 2. Thus, 256 subjects were required. Based on an estimated 10% dropout rate, it was planned to recruit 286 subjects.

To analyze the efficacy data, the full analysis set (FAS) was used, namely, all patients who took the study medication and completed at least one efficacy evaluation. Per protocol (PP) analysis was performed for evaluation of changes in MFR and PVR relative to baseline. Categorical data were compared with chi-square statistics or Fisher's exact test and presented as frequencies. Continuous variables were analyzed using unpaired Student t- test and presented as mean ± standard error. All statistical tests had a two-sided significance level of 0.05 and were performed by using SPSS statistical software package (SPSS 21.0, SPSS, Chicago, IL).

## Results

### 3.1. Baseline demographic data

**Figure 1:**
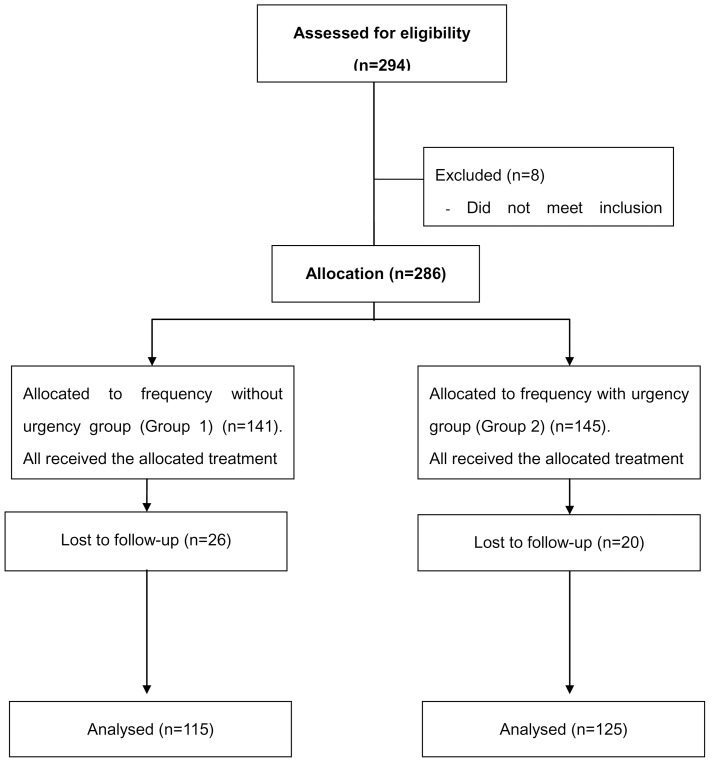
Participant flowchart.

A total of 286 patients were enrolled and 240 (83.9%) successfully completed the 12-week treatment period (Group 1: 115; Group 2: 125) (Figure 1). Twenty-six (18.4%) in Group 1 and 20 (13.8%) Group 2 patients dropped out. The two groups were similar in terms of baseline demographic and clinical characteristics (Table 1). Compliance for solifenacin at 12 weeks was 97.5% and there was no significantly difference between the two groups (97.0% vs. 98.0%, *p* = 0.408).

**Table 1:** Demographics and baseline characteristics.

Variables	Frequency without urgency group (Group 1, n = 115)	Frequency with urgency group (Group 2, n = 125)	*p*-value
**Age, years, mean (range)**	57.46 (26–86)	56.60 (22–82)	0.60
**Sex, n (%)**			0.14
Men	26 (22.61)	19 (15.20)	
Women	89 (77.39)	106 (84.80)	
**Symptom duration, months, mean (range)**	56.46 (3–480)	65.93 (3–720)	0.42
**Voiding diary/24 hour**			
Frequency, mean (range)	12.32 (8.33–22.67)	13.16 (8–32)	0.09
Nocturia, mean (range)	1.86 (0–10.33)	1.74 (0–6)	0.45
Bladder volume, ml, mean (range)	138.34 (32.22–292.31)	136.78 (42.5–315.56)	0.83
**OABSS grade, n (%)**			
Mild	80 (69.57)	15 (12.00)	<0.0001
Moderate	32 (27.83)	89 (71.20)	
Severe	3 (2.61)	21 (16.80)	
**Uroflowmetry parameters**			
Maximal flow rate, ml/s, mean (range)	19.24 (10.4–61.6)	21.53 (10.3–56.5)	0.12
Voided volume, ml, mean (range)	229.75 (122.5–871.5)	215.72 (122.7–831)	0.45
Post-voided residual, ml, mean (range)	25.18 (0–92)	23.35 (0–99)	0.65

### 3.2. Treatment efficacy

At 12 weeks, Groups 1 and 2 both exhibited statistically significant reductions in average daily micturition frequency relative to baseline (−2.49±0.35 and −2.63±0.37; all *p*<0.001); Table 2). There was no significantly difference between two groups. However, the lower limit of the 95% two-sided CI of the comparison of the two group means was −1.14, which is smaller than the −0.8 margin of clinical equivalence (non-inferior p-value  = 0.099). Therefore, it cannot be said that the efficacy of solifenacin for frequency alone was non-inferior to the efficacy of solifenacin for frequency with urgency.

**Table 2:** Non-inferior analysis of solifenacin in patients with urinary frequency with or without urgency.

Variables	Frequency without urgency group (Group 1)	Frequency with urgency group (Group 2)	*p*- value
Baseline	12.32±0.31	13.16±0.38	0.09
Week 12	9.82±0.36	10.52±0.42	0.21
Change from baseline	−2.49±0.35	−2.63±0.37	0.78
95% CI	−3.17, −1.81	−3.37, −1.90	-
95% CI for difference	−3.37, −1.90		

The two groups did not differ significantly in improvement in OABSS, PPBC, or BSW scores (Table 3).

**Table 3:** Patients' perception of treatment benefit, satisfaction, and willing to have retreatment and Changes in Patients' Perception of Bladder Condition scores.

Variables	Frequency without urgency group (Group 1, n = 115)	Frequency with urgency group (Group 2, n = 125)	*p*-value
**Treatment benefit, satisfaction, and willing to have retreatment**			
Benefit	69.9%	73.8%	0.55
Satisfaction	66.3%	75.7%	0.15
Retreatment	83.1%	88.8%	0.26
**Change in Patients**' **Perception of Bladder Condition scores**			
Deterioration	6.0%	6.5%	0.84
No change	16.9%	17.6%	0.72
1-point improvement	30.1%	24.1%	0.41
≥2-point improvement	47.0%	51.9%	0.51

After 4 weeks of treatment, nine patients (7.8%) in Group 1 and four (3.2%) in Group 2 requested a dose escalation to 10 mg. This difference was not statistically significant. In Group 1, the solifenacin escalators had significantly more micturition episodes at baseline than the non-escalators (12.10±1.07 *vs*. 14.81±1.56, *p* = 0.019). The solifenacin escalators and non-escalators in Group 2 did not differ significant in terms of this variable.

### 3.3. Safety

Adverse events were reported in 25.5% (36/141) in Group 1 and 39.3% (57/145), and the difference was not significant. However, these adverse events were generally mild (Table 4). Dry mouth was the most frequently reported adverse reaction. The two groups did not differ significantly in terms of MFR and PVR changes relative to baseline (−1.4±1.05 *vs.*−0.11±0.92 ml/s and 5.5±2.99 *vs.* 9.3±3.94 ml, respectively).

**Table 4:** Treatment-emergent adverse events in both groups.

Variables	Frequency without urgency group (Group 1, n = 115)	Frequency with urgency group (Group 2, n = 125)
**Subjects with AEs, n (%)**	36 (25.5)	57 (39.3)
Dry mouth	13 (36.1)	32 (56.2)
Mild	11	25
Moderate	2	6
Severe	-	1
**Constipation**	2 (5.6)	5 (8.8)
Mild	1	4
Moderate	1	1
Severe	-	-
**Blurred vision**	1 (2.8)	4 (7.0)
Mild	1	4
**Dyspepsia**	6 (16.6)	5 (8.8)
Mild	4	4
Moderate	2	1
**Dizziness**	1 (2.8)	1 (1.7)
Moderate	1	1
**Voiding difficulty**	7 (19.4)	8 (14.1)
Mild	6	6
Moderate	1	2
**Headache**	1 (2.8)	-
Mild	1	
**Fatigue**	2 (5.6)	1 (1.7)
Mild	2	1
**Itching**	3 (8.3)	1 (1.7)
Mild	3	1

## Discussion

To the best of our knowledge, this is the first trial to evaluate the efficacy of an antimuscarinic agent for frequency without urgency. We demonstrated a reduction of approximately two frequency episodes per day in patients with frequency without urgency at week 12, which was similar to that in patients with frequency with urgency, as well as the mean change in daily micturition of antimuscarinic treatment in meta-analysis [10]. The PPBC and BSW questionnaires revealed that the treatment had positive effects in the patients with frequency without urgency, which indicates that the treatment induced clinical meaningful improvements in micturition frequency in this group.

Urinary urgency is the cornerstone of the definition of OAB that is estimated to affect 10% of the worldwide population [11], however yet we know little about the definite cause, effect on voiding behavior, or proper measurement of urgency. OAB patients might modify voiding behavior to avoid showing urgency or urgency incontinence. Therefore, their episodes of urgency may be underestimated or hidden behind frequency only. This can lead to confusion in diagnosis and treatment decision of OAB.

At present, the pathophysiology of frequency without urgency is poorly understood. The most common cause of frequency only would be that the vast majority of patients do not understand the subtle diagnostic nuances of urinary urgency. Indeed, a study on patient understanding of lower urinary tract symptoms showed that the term “urgency” was correctly defined by only 46% of the subjects [12]. A study examining how patients described their urge or desire to urinate reported diverse descriptions, including frequency, full bladder, relief, necessity, and must go [13]. Although the USS has good content validity, discriminated validity, and test-retest reliability [14], it may not capture the patient's experience of urinary urgency thoroughly as other current urgency scales [15]. It may be that these patients void frequently with no preceding desire to void to avoid more compelling situations; this is known as convenience voiding [16]. Healthy volunteers may empty their bladders early for social reasons, such as before joining a meeting, going out on a long journey, or retiring to bed at night [17]. Specifically, OAB patients go to the toilet more often to avoid urgency or leakage because they know of their sudden urgency or urgency incontinence. In these patients, once this decision to convenience void is made, most voiding may be initiated with mild sensations rather than sensation of immediate needing to void. The third explanation of frequency only is the lack of the progressive increase in bladder awareness [18]. These subjects report frequent voiding, which appears to originate from their cognitive strategies to avoid intense urge sensation and incontinence. Thus, interpreting bladder awareness may be depressed in pathological states. Further study is necessary to confirm this modified view for determining the decision to void.

The clinically significant efficacy of an antimuscarinic agent in our patients with frequency without urgency may be explained by an inhibitory effect of this agent on afferent bladder nerves. Antimuscarinic treatment is believed to repress the detrusor overactivity associated with OAB by blocking the muscarinic receptors in the detrusor muscle. However, several studies [19]–[21] have shown that antimuscarinic treatment also significantly improves sensory functions, as shown by increased time to first sensation to void and reduced voiding frequency. On the basis of these observations, we proposed that solifenacin treatment may improve the frequency symptoms of subjects without urgency.

Overall, the 12-week solifenacin treatment was well tolerated in the patients with frequency with and without urgency.

One limitation of this study was that we did not find that solifenacin had a non-inferior effect on frequency without urgency relative to its effect on frequency with urgency in terms of change in daily micturition frequency (the primary endpoint of this study). However, our study did show that solifenacin treatment significantly decreased the daily micturition frequency in patients with frequency regardless of urgency. Moreover, the difference between the urgency and non-urgency groups in terms of micturition frequency change did not achieve statistical significance. The inability of this study to detect non-inferiority may reflect the wide standard deviation (−2.56) associated with the mean number of micturitions per 24 hour that was reported by the study that was used to establish the margin of clinical equivalence for the present study [9]. The second limitation was that there was no placebo group. The placebo response for OAB symptoms is a well-known occurrence during drug trials. Although this was not a placebo-controlled study, we tried to eliminate the effects of the placebo response by recording the voiding diary during the screening period. The placebo response during drug trials in OAB can be partly attributable to the bladder training effect of completing a voiding diary [22]. Through the process of recording and reviewing the voiding diary, patients can become more aware of voiding frequency and they may establish more appropriate voiding intervals. At the initial screening visit, the lifestyle modification (avoidance of bladder irritants such as caffeine), bladder training, and voiding diary were explained, and during the 3 days before the next visit, patients recorded episodes in a 3-day voiding diary. Patients were then assigned to one of two groups. And, a total of 256 subjects enrolled could not reach 80% power to detect non-inferiority because of unexpected subject dropout. We assumed that, although we showed that solifenacin tended to be effective for frequency regardless of urgency, we could not have shown non-inferiority because of low power.

## Conclusions

It was not possible to verify that the solifenacin efficacy for frequency alone was non-inferior to its efficacy for OAB. Nevertheless, solifenacin tended to be effective for frequency regardless of urgency.

## Supporting Information

Checklist S1 CONSORT checklist.
(DOC)

Protocol S1 The original study protocol in Korean.
(DOC)

Protocol S2 English translation of the original study protocol.
(DOC)
