# Supplementary material for: A Comparative Study on the Efficacy of Solifenacin Succinate in Patients with Urinary Frequency with or without Urgency
Source: PLoS One. 2014 Nov 17;9(11):e112063. doi: 10.1371/journal.pone.0112063 (PMC4234319; doi:10.1371/journal.pone.0112063)
Supplement: Protocol S1 — The original study protocol in Korean. (DOC) [file pone.0112063.s002.doc]

**
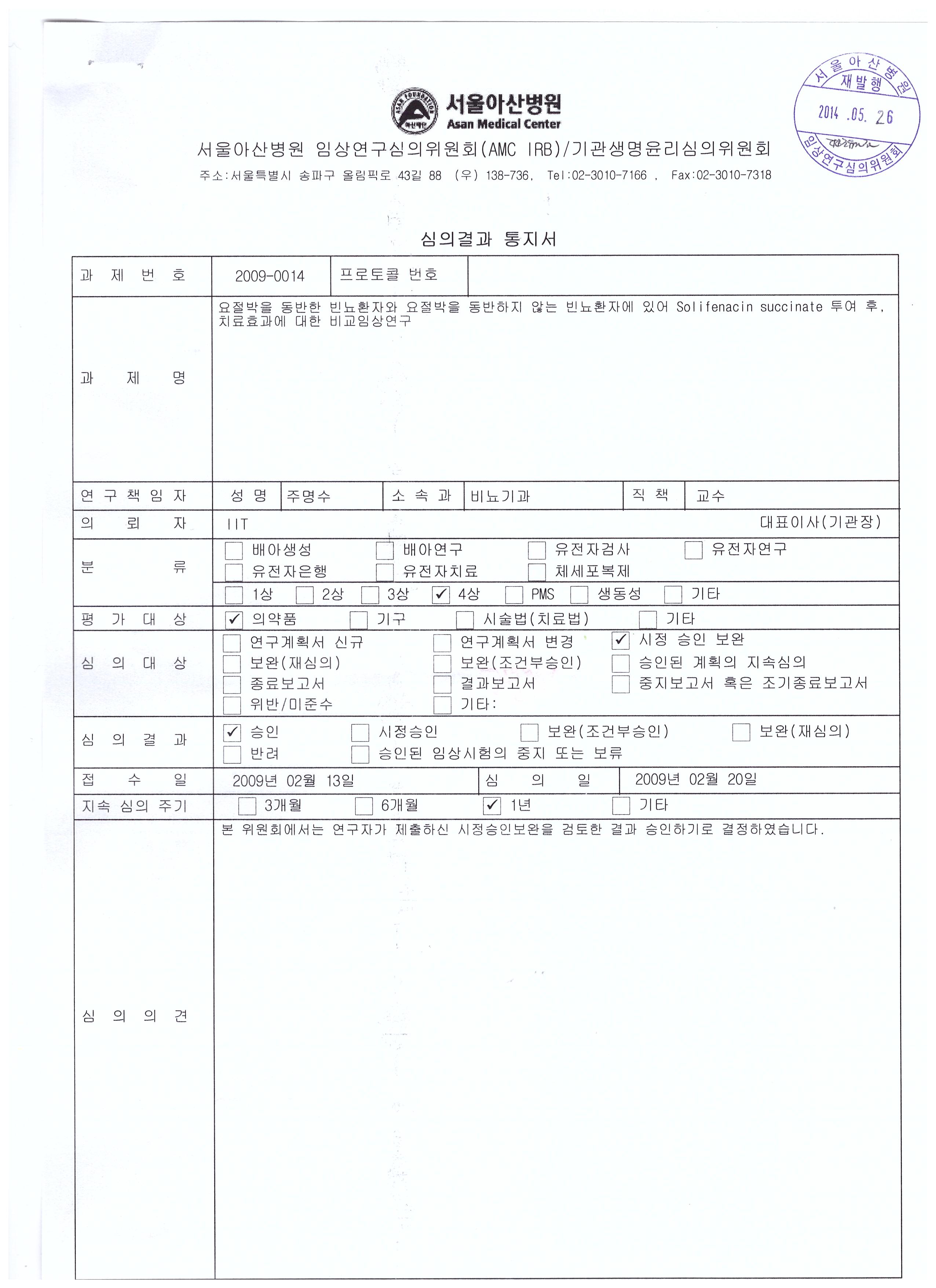
**

**
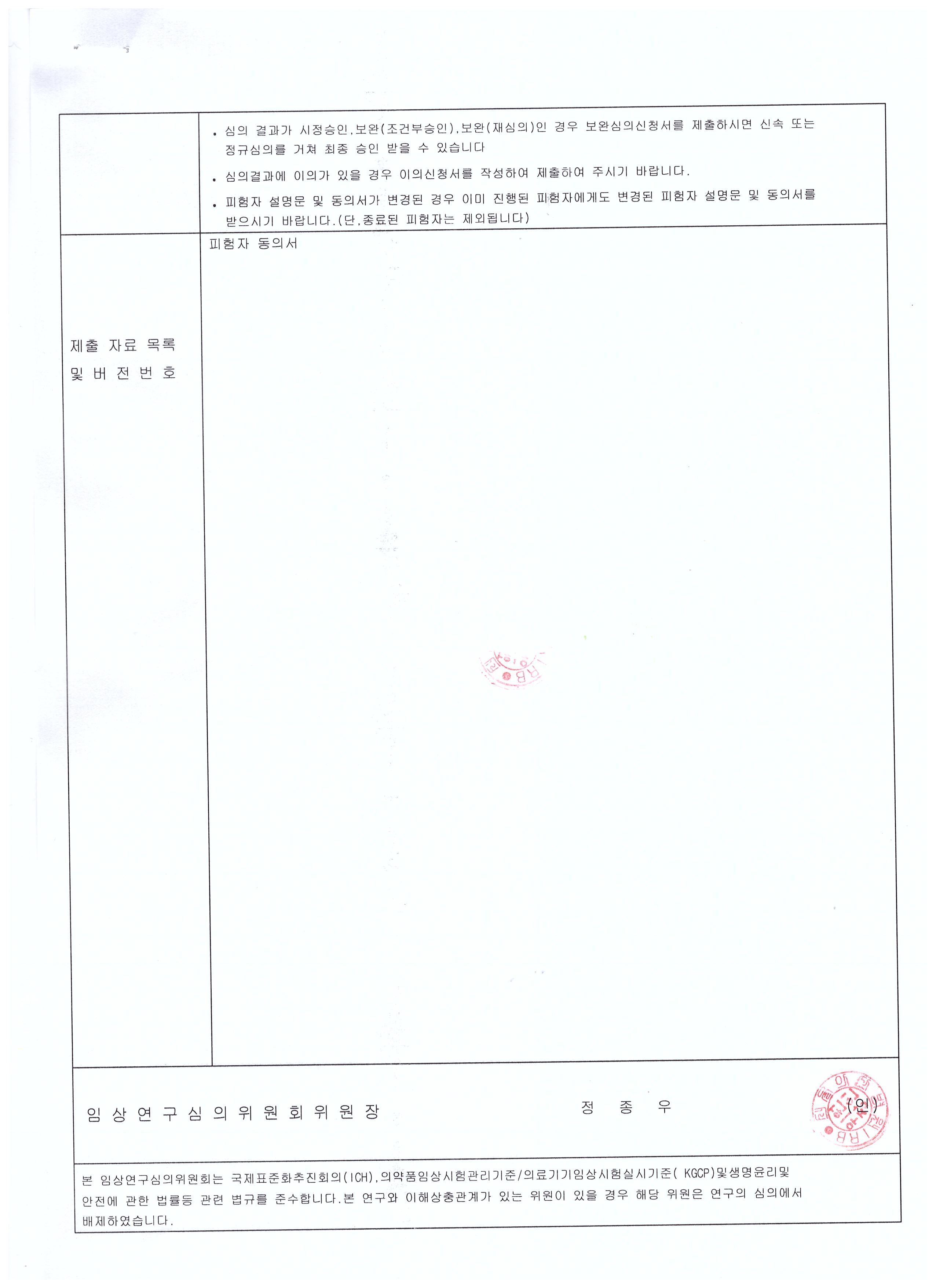
**

This study was approved by the Institutional Review Board of Asan Medical Center on February 20, 2009 (No. 2009-0014) .

**임상연구 프로토콜**

**연구 주제**

# **요절박을 동반한 빈뇨환자와 요절박을 동반하지 않는 빈뇨환자에 있어 Solifenacin succinate 투여 후, 치료효과에 대한 비교임상연구**

# 개요

| **제목** | 요절박을 동반한 빈뇨환자와 요절박을 동반하지 않는 빈뇨환자에 있어 Solifenacin succinate 투여 후, 치료효과에 대한 비교임상연구 |
| --- | --- |
| **후원** | 한국아스텔라스제약 |
| **책임 연구자** | 서울아산병원 비뇨기과 교수 주명수 |
| **목표** | **1. 일차 목표**  그룹 1(요절박이 없는 빈뇨환자군)과 그룹 2(요절박이 있는 빈뇨환자군)를 대상으로 12주간 숙신산 솔리페나신을 투여했을 때 나타나는 배뇨횟수의 변화를 3일간의 배뇨일지를 통해 알아본다.  **2. 이차 목표**   - 숙신산 솔리페나신 치료 12주째에 PPBC(Patient’s Perception of Bladder Condition)의 개선도를 비교한다. - 숙신산 솔리페나신 치료 12주째에 OABSS(OAB Symptom Score)의 개선도를 비교한다. - 숙신산 솔리페나신 치료 12주째에 평균 배뇨량의 변화율을 비교한다. - 숙신산 솔리페나신 치료 12주째에 최대 방광용적의 변화율을 비교한다. - 숙신산 솔리페나신 치료 12주째에 BSW(Benefit, Satisfaction, willingness to continue)설문에 의한 치료만족도를 평가한다. - 그룹 1에서 빈뇨의 호전과 관련된 치료 전 요인들(연령, 성별, 기준시점에서 빈뇨의 발현, 교육수준 등) |
| **평가** | **일차 유효성 평가변수**   - 3일간의 배뇨일지   **이차 유효성 평가변수**   - PPBC (Patient’s Perception of Bladder Condition) 설문 - OABSS(OAB Symptom Score) 설문 - BSW( Benefit, Satisfaction, willingness to continue ) 설문   **안전도 평가 변수**   - 이상반응(adverse events) - 최대 요속 및 잔뇨량 검사 |
| **설계** | Multicenter, comparative, open label, phaseⅣ trial  Non-inferior study  가설: 빈뇨만 있는 환자에게 미치는 솔리페나신의 효과는 요절박이 있는 빈뇨 환자에게 미치는 효과에 비해 열등하지 않다.(non-inferior) |
| **시험약물** | 숙신산 솔리페나신(Vesicare®) |
| **선정기준** | 1. 18세 이상의 남성 또는 여성 2. 24시간당 배뇨횟수 8회 이상(≥ 8/24시간) 3. 배뇨일지에 따라   그룹 1 – 요절박 없음(5점 요절박 척도에서 1~2)  그룹 2 –하루 2회 이상 요절박(≥ 2/day) (5점 요절박 척도에서 3~5)  4. 상위의 증상들이 3개월 이상 지속된 환자 |

| **연구 모집단 기관의 수** | 예상 등록 피시험자의 수: 286명  시험에 끝까지 남을 것으로 예상되는 피시험자의 수: 256명 (그룹당 128명)  (10%의 중도 탈락률에 의거함)  투약기간: 3개월  기관의 수: 9개 센터 |
| --- | --- |
| **통계분석** | 유효성 분석은 FAS 및 PP 분석군을 대상으로 수행된다. 안전성 분석은 한 번 이상 시험약물을 복용한 적이 있는 환자들을 대상으로 진행된다.  - 1차 유효성 평가  군간 배뇨일지(별첨 1)에 따른 배뇨빈도의 변화(기준시점과 종료시점을 비교)를 t검정을 토대로 비교하고, 군간 차이에 대한 95%신뢰구간을 계산한다. 또한, 필요 시 군간 이질성을 보정하기 위하여 일반화된 선형모형(generalized linear model)을 이용한 분석을 시행한다.  - 2차 유효성 평가  PPBC, OABSS 및 BSW(Benefit,Satisfaction,Willingness to continue)등의 2차 유효성 평가변수들에 대해 연속형 자료의 경우 윌콕슨 부호순위 합 검정 또는 paired t 검정 등을, 범주형 자료의 경우 McNemar 검정 등을 이용하여 분석한다. 필요 시 군간 차이에 대한 검정을 위해 변량효과모형(mixed effect model) 또는 일반화 추정방정식(generalized estimation equation)을 이용한 분석을 시행 한다. |
| **예상 시험기간** | 2009년 2월에 환자 스크리닝(적격 심사)을 시작한다.  2009년 6월까지 환자 등록을 마감한다. |

# 연구일정

|  | **V0**  **(– 21 ~0일)** | **V1**  **(0일째)** | **V2**  **(4주±7일)** | **V3**  **(12주±7일)** |
| --- | --- | --- | --- | --- |
|  | **(baseline) *** | |  |  |
| **피험자 동의서** | **●** |  |  |  |
| **인구학적 정보** | **●** |  |  |  |
| **병력, 연관 질환** | **●** |  |  |  |
| **실험실 시험**  CBC  Chemistry**  Urinalysis  PSA (남성에 한함)^***^ | **●**  **●**  **●**  **●** |  |  |  |
| **배뇨일지 배포** | **● ****** | **●** | **●** |  |
| **배뇨일지 수집** |  | **●** | **●** | **●** |
| **PPBC** | **●** |  | **●** | **●** |
| **OABSS** | **●** |  | **●** | **●** |
| **Flow rates (Uroflowmetry)** | **●** |  |  | **●** |
| **잔뇨량 (방광 스캔)** | **●** |  |  | **●** |
| **BSW 설문** |  |  |  | **●** |
| **약물처방(Prescription)** |  | **●** | **●** |  |
| **순응도(Compliance)** |  |  | **●** | **●** |
| **이상반응(Adverse events)** |  |  | **●** | **●** |
| **병용약물** | **●** | **● ******* | **●** | **●** |

각 Visit별 여유 interval은 ± 7일임

* 스크리닝 테스트는 V1에서 약물처방 전에 실시 및 확인되어야 한다.

** Chemistry: AST, ALT, BUN, Cr, ALP, T-bil

*** PSA 수치는 최근 3개월 이내 검사 결과(타기관에서 시행한 검사 결과 사용 가능)

**** 배뇨일지는 V1, V2, V3 전 3일 동안 작성되어야 한다.

***** V1 전에 적어도 2주 동안은 허용되지 않은 약물을 복용하지 않았어야 한다.

**목차**

[요절박을 동반한 빈뇨환자와 요절박을 동반하지 않는 빈뇨환자에 있어 Solifenacin succinate 투여 후, 치료효과에 대한 비교임상연구 1](#__RefHeading___Toc228000646)

[개요 2](#__RefHeading___Toc228000647)

[연구일정 4](#__RefHeading___Toc228000648)

[1. 개요 7](#__RefHeading___Toc228000649)

[2. 목적 7](#__RefHeading___Toc228000650)

[2. 1. 일차 목적 7](#__RefHeading___Toc228000651)

[2. 2. 이차 목적 7](#__RefHeading___Toc228000652)

[3. 연구내용 8](#__RefHeading___Toc228000653)

[4. 환자 선별 9](#__RefHeading___Toc228000654)

[4. 1. 계획된 환자의 수 9](#__RefHeading___Toc228000655)

[4. 2. 기관별 환자의 수 9](#__RefHeading___Toc228000656)

[4. 3. 시험그룹 9](#__RefHeading___Toc228000657)

[4. 4. 피험자 선정기준 9](#__RefHeading___Toc228000658)

[4. 5. 피험자 제외기준 10](#__RefHeading___Toc228000659)

[4. 6. 병용투여의 금지 11](#__RefHeading___Toc228000660)

[5. 치료 11](#__RefHeading___Toc228000661)

[5. 1. 치료제 투약 11](#__RefHeading___Toc228000662)

[5. 2. 허용되는 병용치료 12](#__RefHeading___Toc228000663)

[5. 3. 허용되지 않은 병용치료 12](#__RefHeading___Toc228000664)

[전기자극치료, 자기장치료 12](#__RefHeading___Toc228000665)

[6. 연구약품의 평가 12](#__RefHeading___Toc228000666)

[6. 1. 일차 유효성 평가변수 12](#__RefHeading___Toc228000667)

[6. 2. 이차 유효성 평가변수 12](#__RefHeading___Toc228000668)

[6. 3. 안전도 평가 변수 12](#__RefHeading___Toc228000669)

[6.4 기타 12](#__RefHeading___Toc228000670)

[7. 연구의 수행 13](#__RefHeading___Toc228000671)

[7. 1. Visit 0(- 21~0일) 13](#__RefHeading___Toc228000672)

[7. 2. Visit 1(0일째) 13](#__RefHeading___Toc228000673)

[7. 3. Visit 2(4주째) 13](#__RefHeading___Toc228000674)

[7. 4. Visit 3(12주째) 14](#__RefHeading___Toc228000675)

[8. 이상반응 14](#__RefHeading___Toc228000676)

[8. 1. 정의 14](#__RefHeading___Toc228000677)

[8. 2. 절차 15](#__RefHeading___Toc228000678)

[9. 이탈 15](#__RefHeading___Toc228000679)

[9. 1. 이탈의 사유 15](#__RefHeading___Toc228000680)

[9. 2. 절차 16](#__RefHeading___Toc228000681)

[10. 통계분석 16](#__RefHeading___Toc228000682)

[10. 1. 표본크기의 결정 16](#__RefHeading___Toc228000683)

[10. 2. 분실, 미사용, 불일치 데이터의 취급절차 17](#__RefHeading___Toc228000684)

[10.3. 결과 분석의 일반적 원칙 17](#__RefHeading___Toc228000685)

[10. 4. 통계분석 방법 18](#__RefHeading___Toc228000686)

[11. 연구 윤리 18](#__RefHeading___Toc228000687)

[11. 1. Texts in force 18](#__RefHeading___Toc228000688)

[11. 2. 환자 정보 및 동의 19](#__RefHeading___Toc228000689)

[11. 3. 윤리위원회 제출 19](#__RefHeading___Toc228000690)

[11. 4. 데이터의 기밀성 19](#__RefHeading___Toc228000691)

[12. 참고자료 19](#__RefHeading___Toc228000692)

# **1. 개요**

항무스카린제제는 현재, 과민성 방광(요절박을 주증상으로 절박성 요실금을 동반하거나 동반하지 않으며 통상 빈뇨나 야뇨를 동반하는 증상증후군)의 주처방약제로 사용되어 왔다. 비록 요절박이 과민성방광의 주요 증상이지만 빈뇨 증상 또한, IMPACT와 STARGATE 연구에서 밝혀진 봐와 같이 과민성방광 환자에게서 가장 흔하고 환자를 가장 성가시게 하는 증상으로 판명되었다.

이러한 빈뇨증상은 여러 가지 원인에 의해 발생될 수 있는데, 대표적인 원인으로 요절박, 방광염, 다뇨, 신경정신학적인 요인,생활습관적인 요인 등을 꼽을 수 있다. 요절박은 실제 임상에서 환자나 의사 모두 인지하고 표현하기가 매우 어려운 증상이다. 몇몇 환자의 경우에는 요절박과 일반적인 요의를 구별하지 못하는 경우도 있다. 일부 환자들은 요절박을 느끼기 전에 치골상부에 불쾌감을 느껴 화장실에 가는 경우가 있으며 이 경우 환자들은 요절박의 증상을 갖고 있다는 것을 부인하게 된다. 이런 관점에서, 방광염, 다뇨, 신경학적 요인들을 제외한다면, 요절박를 동반하지 않는 환자라 하더라도 모두 혹은 많은 환자들이 실제로는 과민성방광 환자로 분류 될 수 있다고 예상한다. 이러한 측면에서, 우리는 요절박을 동반하지 않는 빈뇨 환자 대부분에서 항콜린제제에 의해 증상이 개선될 수 있다고 생각한다.

# **2. 목적**

숙신산 솔리페나신이 빈뇨 환자들에게 미치는 효과를 요절박 유무에 따라 비교 고찰한다.

2. 1. 일차 목적

그룹 1(요절박이 없는 빈뇨)과 그룹 2(요절박이 있는 빈뇨)에서 기준시점부터 12주째까지 숙신산 솔리페나신 치료에 따른 배뇨 빈도의 변화를 3일간의 배뇨일지를 토대로 비교한다.

2. 2. 이차 목적

- 숙신산 솔리페나신 치료 12주째에 PPBC(Patient’s Perception of Bladder Condition) 개선도를 비교한다.

- 숙신산 솔리페나신 치료 12주째에 OABSS(OAB Symptom Score)의 개선도를 비교한다.

- 숙신산 솔리페나신 치료 12주째에 평균 배뇨량의 변화율을 비교한다.

- 숙신산 솔리페나신 치료 12주째에 최대 방광용적의 변화율을 비교한다.

- 숙신산 솔리페나신 치료 12주째에 BSW(Benefit, Satisfaction, willingness to continue) 설문을 통해 환자만족도를 측정한다.

- 그룹 1에서 빈뇨의 개선과 관련된 치료 전 요인들(연령, 성별, 기준시점 빈뇨 발현, 교육수준 등)을 파악한다.

# **3. 연구내용**

본 연구는 한국에서 9명의 비뇨기 전문의에 의해 3개월간 수행된 개방형 다기관 비교 관찰 연구이다.

또한 본 연구는 "빈뇨만 있는 환자들에 대한 솔리페나신의 효과는 요절박을 동반하는 빈뇨 환자들과 비교시 열등하지 않다"는 가설에 의거한 비열등성 연구이다.

주명수 교수(책임 연구자) 울산의대 서울아산병원

박원희 교수(공동 연구자) 인하대학교병원

박철희 교수(공동 연구자) 계명대학교 동산의료원

이정구 교수(공동 연구자) 고려대학교 안암병원

이정주 교수(공동 연구자) 부산대학교병원

김덕윤 교수(공동 연구자) 대구 카톨릭대학교병원

이규성 교수(공동 연구자) 성균관대학교 삼성서울병원

나용길 교수(공동 연구자) 충남대학교 병원

권동득 교수(공동 연구자) 전남대학교 병원

0주째부터 3주째까지 스크리닝 기간이다. 시험에 포함된 환자들은 3개월 동안 숙신산 솔리페나신 한 정씩(5mg~10mg)을 받게 된다.

방문은 다음과 같이 네 차례로 진행된다: 대상환자 선정 전 방문(V0), 대상환자 선정 방문(V1), 약물투여 중간시점 방문(V2, 4주 간격), 임상시험종료 방문(V3, 12주째).

필요한 경우에는, V1 방문부터 계산한 이론상의 날짜를 기준으로 ± 7일 전후에 방문이 이루어질 수도 있다.

인구학적 정보에는 체중, 연령, 성별, 교육수준, 직업, 출산경력(여성인 경우) 등이 포함된다.

환자들은 기준시점과 치료 개시 후 4주째와 12주째에 3일간의 배뇨일지를 작성한다.

환자들은 그룹 1(요절박이 없는 빈뇨)과 그룹 2(요절박이 있는 빈뇨)의 두 그룹으로 분류된다.

두 그룹 모두 12주 동안 숙신산 솔리페나신을 복용하게 된다.

- 처음에 환자들은 숙신산 솔리페나신 5mg를 받는다.

- 환자들은 4주째에 연구자와 협의 후 복용량을 10mg으로 증가시킬 수 있다.

- 만약 복용량 증가 후에 환자들이 증가된 양을 견뎌내지 못하면 복용량을 줄일 수 있다.

기준시점과 12주째에는 요속검사 및 잔뇨측정이 실시될 것이다.

환자들은 기준시점과 치료 시작 후 4주째와 12주째에 PPBC(방광상태에 대한 환자의 인식) 및 OABSS설문을 작성한다.

연구 종료 시에는 BSW (Benefit, Satisfaction, willingness to continue) 설문이 작성된다.

# **4. 환자 선별**

4. 1. 계획된 환자의 수

한국에서 286명의 환자들이 포함될 것이다.

그 중 최소한 256명이 3개월간의 약물치료 과정에 끝까지 참여할 것이다.

4. 2. 기관별 환자의 수

기관마다 최소한 32명의 환자가 등록되어 29명이 시험을 마무리해야 한다.

4. 3. 시험그룹

배뇨일지에 따라 모든 환자는 두 그룹에 배정된다. 그룹 1은 요절박이 없는 빈뇨 환자들이고 그룹 2는 요절박을 동반하는 빈뇨 환자들(과민성방광 환자들)이다.

3일 동안 평균 배뇨빈도가 ≥8/24h이고 5점 요절박 척도에서 1~2점을 나타낸 환자들은 그룹 1(빈뇨만 있는 그룹)에 속한다. 3일 동안 ≥8/24h의 평균배뇨빈도와 ≥2/24h의 평균 요절박(5점 요절박 척도에서 3~5점)을 보인 환자들은 그룹 2(빈뇨와 요절박을 동반하는 과민성방광 환자 그룹)로 분류된다.

4. 4. 피험자 선정기준

18세 이상의 남성 또는 여성

그룹 1

- 24시간당 배뇨횟수가 8회 이상인 경우
- 배뇨일지에 요절박이 나타나지 않음(5점 요절박 척도에서 1~2점)

그룹 2

- 24시간당 배뇨횟수가 8회 이상인 경우
- 배뇨일지에서 하루 평균 2회 이상의 요절박 보임(5점 요절박 척도에서 3~5)

상기의 증상이 3개월 이상 지속된 환자

배뇨일지와 설문을 정확하게 작성하려는 능력과 의지가 있는 환자들

치료의 성격 및 그 위험성과 이점을 이해할 수 있고 그에 대한 충분한 논의 후 임상시험 참여 동의서(informed consent form)에 서명한 환자들

4. 5. 피험자 제외기준

아래의 기준들 중 어느 하나라도 해당하는 환자들은 연구에서 제외된다:

1. 연구자의 판단이나 기침유발 검사(여성 환자의 경우)에서 판명된 임상적으로 유의한 복압성요실금이 확인됨.
2. 무작위 배정 전의 배뇨일지에서 일일 평균 총 배뇨량이 3000ml를 초과하는(> 3000 ml) 것으로 확인됨.
3. 아스파라진산 아미노전이효소(AST [SGOT])이나 알라닌 아미노전이효소(ALT [SGPT]), 알칼리성 인산분해효소(alkaline phosphatase), 크레아티닌의 혈청농도에서 기준범위 상한의 두 배를 나타내는 것으로 정의되는 중대한 간질환 또는 신장질환 환자
4. 조절되지 않는 협우각 녹내장이나 요폐(urinary retention) 혹은 위장관 저류(gastric retention)를 포함해 항콜린성 약물치료에 대하여 금기인 질환
5. 준비기간(run-in period) 동안 나타나는 급성 요로감영(UTI) 증상
6. 지난 해에 UTI 증상으로 4회 넘게 치료를 받은 것으로 정의된 재발성 UTI 환자
7. 간질성 방광염(interstitial cystitis)환자로 진단되거나 의심되는 환자
8. 검사되지 않은 혈뇨(hematuria) 또는 악성질환에 부수하는 혈뇨증상
9. 현지 진료기준(잔뇨량 > 100ml)에 따라 임상 증상 및 연구자의 소견에 의해 정의된 임상적으로 유의한 방광출구폐색(bladder outlet obstruction)
10. 현저한 방광류(cystocele)가 있거나 기타 임상적으로 유의한 골반탈출증(pelvic prolapse)이 있는 환자
11. 다음의 치료제로 무작위 배정 전 14일 이내에 치료를 했거나 연구기간 동안 치료를 개시할 것으로 예상되는 경우
    - 임의로 선정된 시험약물 이외의 항콜린성 약물
    - 과민성방광 치료제. 대상환자선정 2개월여 전에 시작된 에스트로겐 치료는 허용된다.
12. 항콜린제의 부작용을 지닌 약제를 불안정 용량으로 복용하고 있거나 연구중 항콜린제의 부작용이 예상되는 환자
13. 무작위 배정 전 14일 이내에 전기자극 또는 방광훈련을 받았거나 연구기간 동안 그러한 치료를 시작을 것으로 예상되는 경우
14. 유치 도뇨관(indwelling catheter)의 사용 또는 간헐적 자가도뇨(intermittent self-catheterization)의 실행
15. 연구 시작 전 1개월 이내에 다른 연구약물을 사용한 경우
16. 만성 변비가 있거나 중증 변비 병력이 있는 환자
17. 임신 또는 수유 중인 여성
18. 성(性)적으로 왕성한 가임기 여성으로 임상시험 개시 전 적어도 1개월 이상 신뢰할 수 있는 피임을 사용하지 않았고 전체 임상시험 기간 동안에 신뢰할 수 있는 피임을 시행하는데 동의하지 않는 환자. 여기서, 신뢰할 수 있는 피임법이란, 자궁내장치, 복합피임약제,호르몬 임플란트,이중막장치,주사용 피임약 및 난관 결찰술이나 정관절제술과 같은 시술을 의미함
19. 방광암이나 전립선암 환자
20. 강력한CYP3A4 저해제 복용 환자(cyclosporine,vinblastine 및erythromycin,

clarithromycin, azithromycinemd과 같은 macrolide계 항생제, ketoconazole,

itraconazole, micronazole과 같은 항진균제)

1. 신경질환 환자
2. 정신질환 환자
3. 연구자의 판단으로 임상연구의 선정기준에 적합하지 않는 환자

4. 6. 병용투여

4.6.1 병용금지

1. 다른 항콜린제
2. 삼환계 항우울제(Tricyclic antidepressants)

단 투약 중단 후 14일의 wash out 기간을 갖고 피험자 등록은 가능

4.6.2 병용 주의 약물

1. 알파 차단제: 임상 시작전 1개월 이상 안정적으로 복용중인 경우
2. 5-알파 reductase inhibitor: 임상 시작전 2개월 이상 안정적으로 복용중인 경우

단 위 두약물을 연구기간 동안에는 지속적으로 복용을 해야 한다.

# **5. 치료**

5. 1. 치료제 투약

치료제: 베이케어정(Vesicare®)

용량: 취침 전에 5~10mg 정제를 하나씩 복용

베시케어정은 씹거나 부수지 않고 한꺼번에 삼켜야 한다.

5. 2. 허용되는 병용치료

금지 약으로 명시된 것 이외의 모든 약은 연구기간 동안 투여될 수 있다.

5. 3. 허용되는 병용치료

전기자극치료, 자기장치료

# **6. 연구약품의 평가**

BSW설문를 제외한 모든 유효성 평가변수는 기준시점과 비교된다.

6. 1. 일차 유효성 평가변수

- 배뇨일지(별첨 1)에 따른 배뇨빈도의 변화(기준시점과 종료시점을 비교)

6. 2. 이차 유효성 평가변수

- PPBC (기준시점과 12주째를 비교)
- OABSS (기준시점과 12주째를 비교)
- 12주째에 BSW(Benefit,Satisfaction,Willingness to continue) 설문

6. 3. 안전도 평가 변수

매 방문 시 자발적으로 보고된 부작용 데이터를 수집하여 전반적인 임상적 안전성을 평가하게 된다. 이를 위해 환자에게 다음과 같은 질문이 제시될 것이다: «”지난번 방문 이후에 특별한 증상이나 전에 없던 건강상의 문제가 있었습니다**?”**»

- 이상반응(adverse events)
- 요속검사를 통해 측정한 최대 요속(MFR)의 변화 (기준시점과 12주째를 비교)
- 방광스캔으로 확인한 배뇨 후 잔뇨량(PVR)의 변화 (기준시점과 12주째를 비교)

전립선비대증 국제자문회의(International Consultation on BPH)의 권고에 따라 기준시점에서 PSA가 측정된다. 표준적인 실험범위가 CRF에서 제공된다.

6.4 기타

혈액 화학검사, 혈액학적 검사, PSA(남성에 한함) 등이 기준시점에서 이루어진다.

# **7. 연구의 수행**

7. 1. Visit 0(- 21~0일)

- 임상시험에 대하여 환자에게 구두로 설명한다.
- 환자가 임상시험 동의서에 날짜를 기입하고 서명한다.
- 인구학적 데이터와 질환병력, 임상병력, 연관 질환, 관련 병용약물 등의 정보를 수집한다.
- 남성에 대한 직장 수지검사(digital rectal exam)를 포함하는 신체검사를 실시한다.
- 실험실 검사: 소변검사, CBC, Chemistry, 혈청 PSA (남자에 한함)
- PPBC, OABSS 평가
- Uroflowmetry 및 잔뇨량 확인
- 설명과 함께 배뇨일지를 환자에게 전달한다.
- 병용 치료제를 확인한다.

7. 2. Visit 1(0일째)

- 실험실 검사 결과를 확인한다.
- 환자들은 배뇨일지에 대한 평가를 토대로 피험자 선정 및 제외기준을 적용하여 임상시험대상환자를 선정한다.
- 베시케어® 정제 5mg 4주 투여량을 처방하고 취침전 복용하게 한다.(약을 씹어서 부수거나 쪼개지 않도록 한다).
- 병용 치료제를 확인한다.
- 환자에게 배뇨일지를 전달한다.

7. 3. Visit 2(4주째)

2차 방문은 포함시점 방문이 있고 나서 4주 후에(필요한 경우 이론상의 날을 기준으로 ±7일 전후에) 이루어질 것이다.

- 부작용을 확인한다.
- 환자가 연구약물을 복용한 날을 확인한다.
- 배뇨일지를 평가한다.
- PPBC 및 OABSS 설문을 평가한다.
- 취침 전에 복용될 베시케어정® 5mg 또는 10mg 8주 투여분을 처방한다(약을 깨물어 부수거나 쪼개지 않도록 한다). 10mg으로의 증량은 연구자와 환자들과의 상의를 통해 결정한다.
- 병용 치료제를 확인한다.
- 환자에게 배뇨일지를 전달한다.

7. 4. Visit 3(12주째)

3차 방문은 대상환자 선정시점 방문이 있고 나서 8주 후에(필요한 경우 이론상의 날을 기준으로 ±7일 전후에) 이루어질 것이다.

- 부작용을 확인한다.
- 환자가 연구약물을 복용한 날을 확인한다.
- 배뇨일지를 평가한다.
- PPBC 및 OABSS설문을 평가한다.
- 병용 치료제를 확인한다.
- Uroflowmetry 및 잔뇨량 확인
- BSW(Benefit, Safety, Willingness to continue) 설문을 통해 연구약물에 대한 환자의 만족도를 평가한다.

# **8. 이상반응**

8. 1. 정의

8. 1. 1. 이상반응의 정의

"임상시험에 사용되는 의약품을 투여 받은 피험자에서 발생한 바람직하지 않고 의도되지 않은 증후(Sign),증상(Symptom),질병을 말하며, 해당 임상시험에 사용된 의약품과 반드시 인과관계를 가져야 하는 것은 아니다."

특히, 투약 중단으로 이어지는 비정상적 실험결과나 증후는 이상반응으로 간주된다.

8. 1. 2. 중대한 이상반응의 정의

임상시험에 사용되는 의약품의 임의의 용량에서 발생한 이상반응 또는 이상약물반응 중에서 다음 중 하나에 해당하는 경우를 말한다.

- 사망을 초래하거나 생명을 위협하는 경우
- 입원 또는 입원기간의 연장이 필요한 경우
- 지속적 또는 의미 있는 불구나 기능 저하를 초래하는 경우
- 선천적 기형 또는 이상을 초래하는 경우
- 기타 의학적으로 중요한 상황

8. 2. 절차

8. 2. 1. 이상반응의 통지 및 문서화

모든 이상반응은 환자 차트에 기록되어야 하고 CRF에 보고되어야 한다.

8. 2. 2. 중대한 이상반응의 통지 및 문서화

연구기간 중(V0부터 V2까지) 언제라도 중대한 이상반응이 관찰되면 연구자는 즉시 또는 근무일 기준 **하루 이내에** 아스텔라스 표준 "중대한 이상반응" 통지를 이용해 전화와 팩스로 그 사실을 알려야 한다.

- 모든 경우에 해당 문서는 연구자의 신원을 밝혀야 하고 날짜와 서명을 포함해야 한다. 또한 부작용과 연구약물 사이의 인과관계에 대한 평가를 제시해야 한다.

8. 2. 3. 연구기간 동안과 그 이후의 절차

연구자는 환자의 상태가 회복되거나 안정될 때까지 임상적 부작용의 결과 또는 비정상적 실험결과를 주시해야 한다.

# **9. 이탈**

9. 1. 이탈의 사유

환자들은 자신의 결정에 따라 언제라도 그 이유와 관계없이 연구에서 이탈할 수 있다. 환자의 이탈은 연구자의 결정에 따라 이루어질 수도 있다. 모든 이탈은 반드시 기록되어야 하고 연구자는 그 이유를 제시해야 한다(예: 환자가 방문하라는 통보를 받은 후에 방문을 이행하지 못함, 환자로부터 협조의 부재, 치료 효능의 부재, 프로토콜에 대한 지속적 준수를 저해하는 임상상태의 악화, 부작용......).

9. 2. 절차

- 연구자는 모든 이탈을 환자 차트와 CRF에 반드시 기록해야 한다.
- 이탈의 원인이 되는 이상반응이 중대한 이상반응의 정의에 부합하면 연구자는 8.2에서 설명한 절차를 이행하게 된다.
- 추적조사에서 소재가 확인되지 않은 환자들의 경우에는 CRF를 그들의 마지막 방문이 이루어진 시점까지 작성해야 한다. 연구자는 환자가 방문하지 못한 이유를 밝히고 환자의 건강상태를 확인하기 위해 최대한 노력할 것이다.

# **10. 통계분석**

10. 1. 표본크기의 결정

표본 크기

각각 128의 그룹 표본크기는 단측 2-표본 T-검정을 통해 80%의 비열등성 검출력을 달성한다. 등가 마진(margin of equivalence)은 -0.80이다. 평균 간의 순수한 차이는 0.00으로 추정된다. 검정의 유의도(알파)는 0.05이다. 데이터는 2.56과 2.56의 표준편차를 보인 환자집단으로부터 얻는다. 따라서 약 286명의 환자가 시험에 등록할 것이며, 이탈률을 10%로 볼 때 그들 중 256명의 환자(치료그룹에서 128명과 통제그룹에서 128명)가 연구에 끝까지 참여할 것으로 예상된다. 샘플 크기는 NCSS 프로그램을 이용해 계산되었다.

결과: NCSS

**Power Analysis of a Non-Inferiority Test of the Difference of Two Means**

**두 가지 평균의 차이에 대한 비열등성 테스트의 검정력 분석**

**비열등성 검정을 위한 수치결과 (H0: D <= -|E|; H1: D > -|E|)**

**검정 통계: T-검정**

**등가 마진 / 실제 차이 / 유의도 / 표준편차 1 / 표준편차 2**

**Power    N1/N2    (E)      (D)      (Alpha)    Beta     (SD1)    (SD2)**

0.80085   325/325   -0.50     0.00     0.05000   0.19915   2.56     2.56

0.80034   226/226   -0.60     0.00     0.05000   0.19966   2.56     2.56

0.80197   167/167   -0.70     0.00     0.05000   0.19803   2.56     2.56

0.80191   128/128   -0.80     0.00     0.05000   0.19809   2.56     2.56

0.80095   101/101   -0.90     0.00     0.05000   0.19905   2.56     2.56

0.80120   82/82      -1.00     0.00     0.05000   0.19880   2.56     2.56

**참고자료**

Chow, S.C.; Shao, J.; Wang, H. 2003. Sample Size Calculations in Clinical Research. Marcel Dekker. New York.

Julious, Steven A. 2004. 'Tutorial in Biostatistics. Sample sizes for clinical trials with Normal data.'

Statistics in Medicine, 23:1921-1986.

10. 2. 분실, 미사용, 불일치 데이터의 취급절차

분석에서 너무 일찍 투약을 중단하게 된 환자들에 대해서는 LOCF(Last Observation Carried Forward) 방법이 사용될 것이다. 이는 기준시점 이후 마지막으로 이루어진 평가(Vend)가 분석된다는(기준시점 값이 이월되지 않는다는) 것을 의미한다.

결측값(missing data)은 자료관리 보고서에서 확인될 것이다.

결측 항목(missing item)을 대체하려는 결정은 연구가 끝나기 전에 이루어진다.

10.3. 결과 분석의 일반적 원칙

본 임상시험의 피험자로부터 얻어진 자료는 크게 ITT (intention to treat) 분석법, FAS (Full data analysis set) 분석법과 PP (per protocol) 분석법의 세가지 형태로 분석된다.

ITT 분석법은 최소한 한 번이라도 임상시험용 의약품을 투여받은 피험자로부터 얻어진 자료를 모두 분석에 포함한다. 이 분석법은 안전성 자료 중 이상반응 자료 분석에 적용된다.

FAS 분석군은 임상시험용 의약품을 투여 받고 1차 유효성 평가 변수를 최소한 한 번이라도 측정한 자료가 있는 피험자로, 그로부터 얻어진 자료는 모두 분석에 포함된다. 단, 선정기준 위반자 및 병용 금기약물 복용자는 분석에서 제외된다. 이때 어떤 시점에서 결측치가 발생되거나 임상시험이 종료되기 전에 피험자가 탈락하면 보수적인 평가를 위하여 가장 최근에 얻은 자료를 마치 해당 시점에서 얻어진 것처럼 자료 분석을 실시한다 (Last Observation Carried Forward Analysis).

PP 분석법은 ITT 분석에 포함되는 피험자 중 임상시험계획서대로 완료한 피험자로부터 얻어진 자료를 분석에 포함시킨다.

유효성에 대한 자료는 원칙적으로 FAS 분석법을 주 분석법으로 하고, PP 분석법으로 추가 분석한다. 안전성에 대한 자료에서 이상반응은 ITT 분석법으로 분석하며 실험실 검사치는 FAS (자료 그 자체) 분석법으로 분석한다. 그 결과를 비교하되 크게 차이를 보이는 경우에는 각 분석법의 결과를 기술한다.

10. 4. 통계분석 방법

10.4.1. 인구통계학적 자료와 병력조사

본 임상시험에 참여한 모든 피험자의 자료를 각 군별로 평가하여 연속형 자료는 평균, 표준편차, 최소, 최대치 등을 구하고 범주형 자료의 경우 절대 및 상대 빈도를 구한다. 두 군간의 인구 통계학적 자료와 기저치 자료를 비교 평가한다. 연속형 변수는 각 군과 시험기관 별로 t-검정 / Mann-Whitney 검정이나 분산분석 검정 / Kruskal-Wallis 검정에 의해 비교한다. 범주형 변수는 카이제곱 검정 및 stratification factor로서 시험기관 별로 Mantel-Haenszel 검정을 이용하여 비교한다. 만일 두 군간의 유의한 차이가 인정되면, 두 군간의 이질성을 보정하기 위해 유효성 분석에서 보정 인자로 사용한다.

10.4.2. 1차 유효성 평가

군간 배뇨일지(별첨 1)에 따른 배뇨빈도의 변화(기준시점과 종료시점을 비교)를 t검정을 토대로 비교하고, 군간 차이에 대한 95%신뢰구간을 계산한다. 또한, 필요 시 군간 이질성을 보정하기 위하여 일반화된 선형모형(generalized linear model)을 이용한 분석을 시행한다.

10.4.2. 2차 유효성 평가

PPBC, OABSS 및 BSW(Benefit,Satisfaction,Willingness to continue)등의 2차 유효성 평가변수들에 대해 연속형 자료의 경우 윌콕슨 부호순위 합 검정 또는 paired t 검정 등을, 범주형 자료의 경우 McNemar 검정 등을 이용하여 분석한다. 필요 시 군간 차이에 대한 검정을 위해 변량효과모형(mixed effect model) 또는 일반화 추정방정식(generalized estimation equation)을 이용한 분석을 시행한다.

# **11. 연구 윤리**

11. 1. Texts in force

시험은 다음에 의거하여 실시된다:

- 헬싱키선언― 1964년 6월 세계의사협회 총회(World Medical Assembly)에서 채택하였으며 1975년 10월 도쿄, 1983년 10월 베니스, 1989년 9월 홍콩, 1996년 10월 서머셋 웨스트에서 각각 수정됨(별첨 3).
- 임상시험관리기준인 Good Clinical Practice를 위한 ICH 지침

11. 2. 환자 정보 및 동의

환자가 시험에 참여하기 전에 시험의 목적과 방법을 설명해준다. 환자는 설명을 듣고 숙지된 사항에 대한 자신의 동의를 서면으로 제공한다.

11. 3. 윤리위원회 제출

연구자/책임자는 규정상 요구되는 서류를 현지 윤리위원회에 제출하고 서면으로 윤리위원회의 의견을 확보한다. 윤리위원회의 승인이 이루어질 때까지 환자들은 연구에 참여할 수 없다.

시험기간 동안 프로토콜에 수정이나 변경이 있으면 그 사항을 윤리위원회에 알려야 한다. 윤리위원회는 환자의 안전성에 영향을 미치거나 시험의 지속을 저해할 가능성이 있는 사건이 있는 경우에도 그에 대한 통보를 받아야 한다.

11. 4. 데이터의 기밀성

데이터 수집 및 처리 중에 기밀유지에 대한 환자의 권리가 유지된다.

# **12. 참고자료**

1. Abrams P, Cardozo L, Fall M, Griffiths D, Rosier P, Ulmsten U, van Kerrebroeck P, Victor A, Wein A. The standardization of terminology in lower urinary tract function: report from the standardization sub-committee of the International Continence Society. Neurourol Urodyn 2002;21:167-78

2. Irwin DE, Milsom I, Hunskaar S, Reilly K, Kopp Z, Herschorn S, Coyne K, Kelleher C, Hampel C, Artibani W, Abrams P. Population-based survey of urinary incontinence, overactive bladder, and other lower urinary tract symptoms in five countries: results of the EPIC study. Eur Urol 2006;50:1306-15

3. Einhoff V, Bavenden T, Glasser DB, Carlsson M, Eyland N, Roberts R. Symptom-specific efficacy of tolterodine extended release in patients with overactive bladder: the IMPACT trial. Int J Cli Pract 2006;60:745-51

4. Choo MS, Doo CK, Lee KS. Satisfaction with tolterodine: assessing symptom specific patient-reported goal achievement in the treatment of overactive bladder in female patients. (STARGATE study) 2008;62:191-6
