# Supplementary material for: A Comparative Study on the Efficacy of Solifenacin Succinate in Patients with Urinary Frequency with or without Urgency
Source: PLoS One. 2014 Nov 17;9(11):e112063. doi: 10.1371/journal.pone.0112063 (PMC4234319; doi:10.1371/journal.pone.0112063)
Supplement: Protocol S2 — English translation of the original study protocol. (DOC) [file pone.0112063.s003.doc]

**Clinical Study Protocol**

**Study Title:**

**A Comparative Clinical Study of the Effects of Solifenacin Succinate Treatment in Patients Who Have Frequent Micturition with and without Urgency**

# SYNOPSIS

| **Title** | A Comparative Clinical Study of the Effects of Solifenacin Succinate Treatment in Patients Who Have Frequent Micturition with and without Urgency |
| --- | --- |
| **Sponsor** | Astellas Korea |
| **Principal**  **Investigator** | Professor Myung-Soo Choo, Asan Medical Center Department of Urology |
| **Objectives** | **1. Primary objective**  To compare changes in micturition frequency on 3-day voiding diary from baseline to week 12- of treatment with solifenacin succinate in group 1 (frequency without urgency) and group 2 (frequency with urgency).  **2. Secondary objectives**   - To compare improvements in the patient’s perception of bladder condition (PPBC) at week 12 of treatment with solifenacin succinate. - To compare improvements in the OAB symptom score (OABSS) at week 12 of treatment with solifenacin succinate. - To compare percent changes in mean void volume at week 12 of treatment with solifenacin succinate. - To compare percent changes in maximum bladder capacity at week 12 of treatment with solifenacin succinate. - To assess patient satisfaction by the Benefit, Satisfaction and Willingness to Continue (BSW) questionnaire at week 12 of treatment with solifenacin succinate. - To identify pre-treatment factors (age, sex, baseline episodes of frequency, education level, etc) associated with frequency improvement in group 1 |
| **Evaluation** | **Primary efficacy endpoint**   - 3-day voiding diary   **Secondary efficacy endpoints**   - Patient’s Perception of Bladder Condition (PPBC) questionnaire - Overactive bladder symptom score (OABSS) - Benefit, Satisfaction and Willingness to Continue (BSW) questionnaire   **Safety endpoints**   - Adverse events - maximum flow rate and post-void residual urine volume |
| **Design** | Multicenter, comparative, open-label phase IV trial  Non-inferiority study  Hypothesis: The effect of solifenacin is not inferior in patients with frequency only to others with both frequency and urgency. |
| **Study drug** | Solifenacin Succinate (Vesicare®) |
| **Inclusion Criteria** | 1. Male or female aged ≥ 18 years 2. Micturition frequency ≥ 8/24 hrs 3. Grouped based on voiding diary data:   For group 1, no urinary urgency (1 or 2 on 5-point urgency scale)  For group 2, ≥ 2 urinary urgency episodes per day (3 to 5 on 5-point urgency scale)   1. Continuous presence of the symptoms above for more than 3 months |

| **Study population and Number of study centers** | Expected number of subjects enrolled: 286 patients  Number of subjects expected to complete the study : 256 patients (128 in each group), considering a10% drop-out rate  Duration of medication: 3 months  Number of centers: 9 centers |
| --- | --- |
| **Statistical analysis** | Efficacy analysis will be performed in FAS and PP sets, whereas safety analysis will be conducted in those patients who have taken the study drug at least once.  - Primary efficacy assessment  A t-test will be used to compare changes in frequency of urination (from baseline to the end of the study) between study groups on the voiding diary (see Annex 1) and calculate a 95% confidence interval for the difference between the groups. Further analysis, if necessary, will be conducted with a generalized linear model in order to correct heterogeneity between the groups.  - Secondary efficacy assessment  Secondary efficacy endpoints (i.e., PPBC, OABSS and BSW) will be analyzed using the the Wilcoxon signed-rank sum test or paired t-test for continuous data and the McNemar test for categorical data. If necessary, a mixed effect model or generalized estimation equation will be used as an analysis tool to test the difference between study groups. |
| **Expected study duration** | Patient screening begins in February 2009.  The last patient enrollment is completed by the end of June 2009. |

# Study Schedule

|  | V0  (Days -21 to 0) | V1  (Day 0) | V2  (Week 4 ± 7 days) | V3  (Week 12 ± 7 days) |
| --- | --- | --- | --- | --- |
|  | (baseline) * | |  |  |
| Informed consent | **●** |  |  |  |
| Demographics | **●** |  |  |  |
| Medical history, associated  diseases | **●** |  |  |  |
| Laboratory test:  CBC  Chemistry**  Urinalysis  PSA (only in male)^***^ | **●**  **●**  **●**  **●** |  |  |  |
| Voiding diary dispensed | **● ****** | **●** | **●** |  |
| Voiding diary collected |  | **●** | **●** | **●** |
| PPBC | **●** |  | **●** | **●** |
| OABSS | **●** |  | **●** | **●** |
| Flow rate (Uroflowmetry) | **●** |  |  | **●** |
| Residual urine (Bladder scan) | **●** |  |  | **●** |
| BSW questionnaire |  |  |  | **●** |
| Prescription |  | **●** | **●** |  |
| Compliance |  |  | **●** | **●** |
| Adverse events |  |  | **●** | **●** |
| Concomitant medications | **●** | **● ******* | **●** | **●** |

Intervals between different visits: ± 7 days

* Screening tests should be performed and confirmed prior to prescription at V1.

** Chemistry: AST, ALT, BUN, Cr, ALP, T-bil

*** PSA leves should be measurements within the last 3 months (replaceable by data from other institutions).

**** Voiding diary should be kept for 3 days prior to V1, V2 and V3.

***** Any prohibited drugs must not have been taken for at least 2 weeks prior to V1.

**Table of Contents**

[SYNOPSIS 2](#__RefHeading___Toc388511531)

[Study Schedule 4](#__RefHeading___Toc388511532)

[1. Introduction 7](#__RefHeading___Toc388511533)

[2. Study Objectives 7](#__RefHeading___Toc388511534)

[2. 1. Primary objective 7](#__RefHeading___Toc388511535)

[2. 2. Secondary objectives 7](#__RefHeading___Toc388511536)

[3. Description of the Study 7](#__RefHeading___Toc388511537)

[4. Selection of Patients 8](#__RefHeading___Toc388511538)

[4. 1. Planned number of patients 8](#__RefHeading___Toc388511539)

[4. 2. Number of patients per center 9](#__RefHeading___Toc388511540)

[4. 3. Study groups 9](#__RefHeading___Toc388511541)

[4. 4. Inclusion criteria 9](#__RefHeading___Toc388511542)

[4. 5. Exclusion criteria 9](#__RefHeading___Toc388511543)

[4. 6. Use of concomitant medications 10](#__RefHeading___Toc388511544)

[4.6.1 Prohibited concomitant medications 10](#__RefHeading___Toc388511545)

[4.6.2 Concomitant medications to be used with care 11](#__RefHeading___Toc388511546)

[5. Treatment 11](#__RefHeading___Toc388511547)

[5. 1. Treatment administration 11](#__RefHeading___Toc388511548)

[5. 2. Permitted concomitant therapy 11](#__RefHeading___Toc388511549)

[5. 3. Non-permitted concomitant therapy 11](#__RefHeading___Toc388511550)

[6. Evaluation of the Study Drug 11](#__RefHeading___Toc388511551)

[6. 1. Primary efficacy endpoint 11](#__RefHeading___Toc388511552)

[6. 2. Secondary efficacy endpoints 11](#__RefHeading___Toc388511553)

[6. 3. Safety endpoints 11](#__RefHeading___Toc388511554)

[6.4 Other 12](#__RefHeading___Toc388511555)

[7. Practical Conduct of the Study 12](#__RefHeading___Toc388511556)

[7. 1.Visit 0 (days -21 to 0) 12](#__RefHeading___Toc388511557)

[7. 2.Visit 1 (day 0) 12](#__RefHeading___Toc388511558)

[7. 3. Visit 2 (week 4) 12](#__RefHeading___Toc388511559)

[7. 4. Visit 3 (week 12) 13](#__RefHeading___Toc388511560)

[8. Adverse Events 13](#__RefHeading___Toc388511561)

[8. 1. Definition 13](#__RefHeading___Toc388511562)

[8. 2. Procedures 13](#__RefHeading___Toc388511563)

[9. Drop-out 14](#__RefHeading___Toc388511564)

[9. 1. Reasons for drop-out 14](#__RefHeading___Toc388511565)

[9. 2. Procedures 14](#__RefHeading___Toc388511566)

[10. Statistical Analysis 14](#__RefHeading___Toc388511567)

[10. 1. Sample size determination 14](#__RefHeading___Toc388511568)

[10. 2. Handling of missing, unused or inconsistent data 15](#__RefHeading___Toc388511569)

[10.3. General principles of outcome analysis 15](#__RefHeading___Toc388511570)

[10. 4. Statistical analysis methods 15](#__RefHeading___Toc388511571)

[11. Ethics 16](#__RefHeading___Toc388511572)

[11. 1. Texts in force 16](#__RefHeading___Toc388511573)

[11. 2. Patient information and consent 16](#__RefHeading___Toc388511574)

[11. 3. Submission to an Ethics Committee 16](#__RefHeading___Toc388511575)

[11. 4. Confidentiality of the data 16](#__RefHeading___Toc388511576)

[12. References 17](#__RefHeading___Toc388511577)

# **1. Introduction**

Antimuscarinic drugs have been a mainstay of treatment for overactive bladder (OAB) which is defined as urgency, with or without urge incontinence, usually with frequency and nocturia by ICS. Although urgency is a key symptom of OAB, frequency is reported as one of the most common and bothersome symptoms in OAB patients, as found in IMPACT and STARGATE studies.

This urinary frequency may be caused by many different factors including urgency, cystitis, polyuria, polysypsia, neuropsychiatric factors and habitual factors. Urgency is a very difficult condition for patients and doctors to perceive and describe. Some patients cannot discriminate urgency from normal urge. Some should go to the toilet because of the discomfort (not pain) on suprapubic area before they feel urgency, in which case they deny the presence of urgency. In this sense, all or many patients who have urinary frequency without urgency may be classified as OAB cases, if cystitis, poyuria and neuropsychiatric factors are not taken into consideration. Consequently, we think that anticholinergics can improve symptoms in most patients who have urinary frequency without urgency.

# **2. Study Objectives**

To examine the effects of solifenacin succinate treatment in patients who have frequent micturition with or without urgency.

2. 1. Primary objective

To compare changes in micturition frequency on 3-day voiding diary from baseline to week 12 of treatment with solifenacin succinate between group 1 (frequency without urgency) and group 2 (frequency with urgency)

2. 2. Secondary objectives

- To compare improvements in the PPBC at week 12 of treatment with solifenacin succinate.

- To compare improvements in the OABSS at week 12 of treatment with solifenacin succinate.

- To compare percent changes in mean void volume at week 12 of treatment with solifenacin succinate.

- To compare percent changes in maximum bladder capacity at week 12 of treatment with solifenacin succinate.

- To assess patient satisfaction by the BSW questionnaire at week 12 of treatment with solifenacin succinate.

- To identify pre-treatment factors (age, sex, baseline episodes of frequency, education level, etc) associated with frequency improvement in group 1.

# **3. Description of the Study**

This study is an open-label, multi-center, observational, comparative trial conducted for three months by nine urologists in South Korea.

This study is also a non-inferiority study hypothesizing that the effect of solifenacin is not inferior in patients with frequency only to others with both frequency and urgency.

Principal investigator: Prof. Myung-Soo Choo, University of Ulsan Asan Medical Center

Co-investigator: Prof. Won Hee Park, Inha University Hospital

Co-investigator: Prof. Chul Hee Park (co-investigator), Keimyung University Dongsan Medical Center

Co-investigator: Prof. Jeong Ku Lee, Korea University Hospital

Co-investigator: Prof. Jeong Zoo Lee, Pusan National University Hospital

Co-investigator: Prof. Duk Yoon Kim, Daegu Catholic University Hospital

Co-investigator: Prof. Kyu-Sung Lee, Samsung Medical Center, Sungkyunkwan University

Co-investigator: Prof. Yong-Gil Na, Chungnam National University Hospital

Co-investigator: Prof. Dong Deuk Kwon, Chonnam National University Hospital

In this study, the screening period is from week 0 to week 3. Patients included in the trial will receive one tablet of solifenacin succinate (5 mg or 10 mg) for a period of three months.

This study will involve four planned visits: pre-inclusion visit (V0), inclusion visit (V1), 1 intermediate visit (V2, 4-week intervals) and end-of-study visit (V3, week 12).

If needed, visits may take place within 7 days before or after the theoretical dates calculated from V1.

Demographics include body weight, age, sex, education level, occupation and parity (for female only). Patients are asked to keep a 3-day voiding diary at baseline and at weeks 4 and 12 after initiation of treatment.

Patients are divided into two groups: group 1 (frequency without urgency) and group 2 (frequency with urgency). Both of the groups receive solifenacin succinate over a period of 12 weeks.

- At the beginning, patients receive solifenacin succinate 5 mg.

- At week 4, they can have the dose escalated to 10 mg after consultation with the investigator.

- If there are patients who are not able to withstand the new dose after dose escalation, it can be reduced back.

Uroflowmetry and residual urine measurement will be performed at baseline and week 12.

Patients will be asked to complete the PPBC and OABSS questionnaires at baseline and at weeks 4 and 12 of treatment. At the end of the study, they will be asked to complete the BSW questionnaire.

# **4. Selection of Patients**

4. 1. Planned number of patients

This study will include 286 patients recruited in South Korea, and at least 256 of them are expected to complete our 3-month medication course.

4. 2. Number of patients per center

Each study center should enroll at least 32 patients, 29 of whom should complete the study.

4. 3. Study groups

Based on voiding diary data, subjects are assigned to one of two groups: Group 1 includes patients who have frequency without urgency, while group 2 consists of those who have frequency with urgency (overactive bladder patients).

Patients in group 1 (frequency-only group) are those with a mean urinary frequency of ≥ 8 micturitions per 24 hours for three days and a score of 1-2 on the 5-point urgency scale. Group 2 (OAB group with frequency and urgency) includes patients who experience on average ≥ 8 micturitions per 24 hours for three days and ≥ 2 urgency episodes per 24 hours (or 3-5 on the 5-point urgency scale) they belong to group 2.

4. 4. Inclusion criteria

Subjects must meet all of the following inclusion criteria to be eligible for enrollment in this study:

Male or female aged ≥ 18 years

For group 1

- Urinary frequency ≥ 8 micturitions per 24 hours
- No urgency on voiding diary (1 or 2 on 5-point urgency scale)

For group 2

- Urinary frequency of ≥ 8 micturitions per 24 hours
- ≥ 2 episodes of urgency on average per day on voiding diary (3-5 on 5-point urgency scale)

Continuous presence of the symptoms above for more than 3 months.

Ability and willingness to complete the voiding diary and questionnaires accurately

Capable of understanding and having signed the informed consent form after full discussion of the research nature of the treatment and its risks and benefits

4. 5. Exclusion criteria

Patients presenting with any of the following will be excluded from this study:

1. Clinically significant stress incontinence as determined by the investigator or confirmed by a cough provocation test for female patients.
2. Total daily urine volume of > 3000 ml as verified on the voiding diary before randomization
3. Significant hepatic or renal disease, defined as having twice the upper limit of the reference ranges for serum concentrations of aspartate aminotransferase (AST [SGOT]), alanine aminotransferase (ALT [SGPT]), alkaline phosphatase or creatinine
4. Any condition that is a contraindication for anticholinergic treatment, including uncontrolled narrow-angled glaucoma, urinary retention or gastric retention
5. Symptomatic acute urinary tract infection (UTI) during the run-in period
6. Recurrent UTIs defined as having been treated for symptomatic UTIs > 4 times in the last year
7. Diagnosed or suspected interstitial cystitis
8. Uninvestigated hematuria or hematuria secondary to malignant disease.
9. Clinically significant bladder outlet obstruction defined by clinical symptoms and in the investigator’s opinion according to the local standard of care (residual urine > 100 ml)
10. Marked cystocele or other clinically significant pelvic prolapse.
11. Treatment within 14 days prior to randomization, or expected to initiate treatment during the study, with:
    - Any anticholinergic drug other than the randomized trial drug
    - Any drug treatment for overactive bladder. Estrogen treatment started more than 2 months prior to inclusion is allowed.
12. On an unstable dosage of any drug with anticholinergic side effects, or expected to start such treatment during the study
13. Receipt of any electrostimulation or bladder training within 14 days before randomization, or expected to start such treatment during the study
14. Use of an indwelling catheter or practicing intermittent self-catheterization
15. Use of any investigational drug within one month preceding the start of the study
16. Chronic constipation or history of severe constipation
17. Pregnant or nursing women
18. Sexually active females of childbearing potential not using reliable contraception for at least one month prior to the start of the clinical trial and not agreeing to use such methods during the entire study period. Reliable contraceptive methods are defined as intrauterine devices (IUDs), combination type contraceptive pills, hormonal implants, double barrier method, injectable contraceptives and surgical procedures such as tubal ligation or vasectomy.
19. Presence of bladder cancer or prostate cancer
20. Patients on treatment with potent CYP3A4 inhibitors, such as cyclosporine, vinblastine, macrolide antibiotics (e.g. erythromycin, clarithromycin and azithromycin) or antifungal agents (such as ketoconazole, itraconazole and micronazole).
21. Presence of any neurological disease
22. Presence of any psychological disease
23. Any other conditions which, in the investigator’s opinion, make the patient unsuitable for inclusion

4. 6. Use of concomitant medications

4.6.1 Prohibited concomitant medications

1. Other anticholinergics
2. Tricyclic antidepressants

Subjects who have discontinued any of these medications must have a wash-out period of 14 days and can be enrolled in the study.

4.6.2 Concomitant medications to be used with care

1. Alpha blockers (which have been stably used for more than one month prior to the start of the study)
2. 5-alpha reductase inhibitors (which have been stably used for more than two months prior to the start of the study)

Patients who are on any of those concomitant medications must continue to take them during the study.

# **5. Treatment**

5. 1. Treatment administration

Treatment: Vesicare®

Posology: One 5 mg or 10 mg tablet to be taken before sleep

The Vesicare tablet should be swallowed whole without being chewed or crushed.

5. 2. Permitted concomitant therapy

All drugs other than those specified as unauthorized drugs are allowed during the study.

5. 3. Non-permitted concomitant therapy

Electrostimulation and magnetic therapy

# **6. Evaluation of the Study Drug**

All efficacy endpoints, except the BSW questionnaire, are compared with baseline.

6. 1. Primary efficacy endpoint

- Changes in micturition frequency on the voiding diary (Appendix 1) (from baseline to end of study)

6. 2. Secondary efficacy endpoints

- PPBC (changes from baseline to week 12)
- OABSS (changes from baseline to week 12)
- BSW questionnaire at week 12

6. 3. Safety endpoints

General clinical safety will be assessed based on data on spontaneously reported adverse events at each visit. To this end, subjects will be asked: “Since last visit*,* have you had any unusual symptoms or any health problems you had never experienced before?”

- Adverse events
- Changes in maximum flow rate (MFR) measured by uroflowmetry (from baseline to week 12)
- Changes in post-void residual (PVR) urine volume assessed by bladder scan (from baseline to week 12)

According to the recommendations of the International Consultation on BPH, the PSA will be measured at baseline. Normal laboratory ranges will be provided in the CRF.

6.4 Other

A blood chemistry examination, hematologic test and PSA (only in male) will be performed at baseline.

# **7. Practical Conduct of the Study**

7. 1.Visit 0 (days -21 to 0)

- An oral explanation of the clinical trial to subjects.
- Dating and signing of the informed consent form by subjects
- Collection of necessary information including demographics, disease history, clinical history and associated conditions and relevant concomitant medications.
- Physical examinations including a digital rectal exam in male subjects.
- Laboratory tests: Urinalysis, CBC, chemistry and serum PSA (only in male subjects)
- PPBC and OABSS assessments
- Uroflowmetry and residual urine assessment
- A voiding diary is explained and given to each subject.
- Identification of concomitant medications

7. 2.Visit 1 (day 0)

- Laboratory test results are confirmed.
- Subjects for the clinical trial are selected based on the inclusion and exclusion criteria after an evaluation of their voiding diaries.
- Subjects are prescribed one Vesicare® 5 mg tablet to take before sleep for four weeks. The tablet should not be chewed, divided or crushed.
- Concomitant medications are identified.
- The voiding diary is given to each subject.

7. 3. Visit 2 (week 4)

Visit 2 will take place four weeks after the inclusion visit (or if necessary, within seven days before or after the theoretical date).

- Adverse events are identified.
- The date when each subject took the study drug is confirmed.
- The voiding diary is evaluated.
- The PPBC and OABSS questionnaires are evaluated.
- Subjects are prescribed one 5 or 10mg Vesicare® 5 mg or 10 mg tablet to take before sleep for eight weeks. The tablet should not be chewed, divided or crushed. Dose escalation to 10 mg is determined after consultation between the investigator and subjects.
- Concomitant medications are identified.
- The voiding diary is given to each subject.

7. 4. Visit 3 (week 12)

Visit 3 will take place eight weeks after the inclusion visit (or if necessary, within seven days before or after the theoretical date).

- Adverse events are identified.
- The date when each subject took the study drug is confirmed.
- The voiding diary is evaluated.
- The PPBC and OABSS questionnaires are evaluated.
- Concomitant medications are identified.
- Uroflowmetry and residual urine assessment
- Patient satisfaction with the study drug is assessed by the BSW questionnaire.

# **8. Adverse Events**

8. 1. Definition

8. 1. 1. Definition of adverse events

“Any unfavorable or unintended sign, symptom or disease occurring in subjects in association with the use of an investigational medicinal product and which does not necessarily have a causal relationship with the investigational drug. ”

In particular, any abnormal laboratory finding or sign which leads to treatment discontinuation is to be considered as an adverse event.

8. 1. 2. Definition of serious adverse events

A serious adverse event is any untoward medical occurrence or adverse drug reaction that at any dose of an investigational drug:

- results in death or is life-threatening;
- requires hospitalization or prolongation of existing hospitalization
- causes a persistent or significant disability or incapacity.
- results in a congenital anomaly or birth defect; or
- is associated with any other medically important condition. .

8. 2. Procedures

8. 2. 1. Notification and documentation of adverse events

Any adverse event should be documented on the patient chart and reported on the case report form (CRF).

8. 2. 2. Notification and documentation of serious adverse events

If any serious adverse event is found during the study (V0 to V2), the investigator must report the occurrence of such event immediately or **within one working day** to Astellas Korea by phone or fax, using Astellas’ standard SAE report form.

- In any case, the report must show the identity of the investigator and be dated and signed. It should also give an assessment of the causal relationship between the event and the study drug.

8. 2. 3. Procedures during and after the study

The investigator should follow up and monitor patients for the outcome of clinical adverse events or abnormal laboratory findings until recovery or stabilization of their condition.

# **9. Drop-out**

9. 1. Reasons for drop-out

Patients may drop out of the study at any time, for whatever reason, once they decide to do so, or the investigator may decide to withdraw patients from the study. All dropouts must be documented and the investigator must specify the reasons (e.g., failure of a patient to pay a visit despite receipt of notice, lack of cooperation from the patient, lack of treatment efficacy, worsening of the clinical condition obstructing continued compliance with the protocol, occurrence of adverse events ...).

9. 2. Procedures

- All dropouts must be recorded on the patient chart and CRF.
- If any event causing a drop-out meets the definition of serious adverse events, the investigator will carry out the procedures as described in section 8.2.
- For patients lost to follow-up, the CRF must be completed until the date the last visit took place. The investigator will use his/her best effort to find out the reason why a patient failed to pay the visit, if any, and to determine the state of his/her health.

# **10. Statistical Analysis**

10. 1. Sample size determination

Sample Size

A sample size of 128 in each group achieves 80% power to detect non-inferiority using a **two**-sided, two-sample t-test. The margin of equivalence is -0.80. The true difference between means is assumed to be 0.00. The significance level (alpha) of the test is 0.05. The data are drawn from patient populations with standard deviations of 2.56 and 2.56. Thus, approximately 286 patients will be enrolled in the trial and 256 patients of them (128 in the treatment group and 128 in the control group) are expected to complete the study assuming a drop rate of 10%. The sample size was calculated using the NCSS program.

Result: NCSS

**Power analysis of a non-inferiority test for the difference between two means**

**Numeric results for a non-inferiority test (H0: D <= -|E|; H1: D > -|E|)**

**Test statistic: T-test**

**Equivalence Margin / Actual Difference / Significance / Standard Deviation 1 / Standard Deviation 2**

**Power N1/N2 (E) (D) (Alpha) Beta (SD1) (SD2)**

0.80085  325/325 -0.50 0.00 0.05000 0.19915 2.56 2.56

0.80034  226/226 -0.60 0.00 0.05000 0.19966 2.56 2.56

0.80197 167/167 -0.70 0.00 0.05000 0.19803 2.56 2.56

0.80191 128/128 -0.80 0.00 0.05000 0.19809 2.56 2.56

0.80095 101/101 -0.90 0.00 0.05000 0.19905 2.56 2.56

0.80120  82/82 -1.00 0.00  0.05000 0.19880 2.56 2.56

**References**

Chow, S.C.; Shao, J.; Wang, H. 2003. Sample Size Calculations in Clinical Research. Marcel Dekker. New York.

Julious, Steven A. 2004. 'Tutorial in Biostatistics. Sample sizes for clinical trials with Normal data.'

Statistics in Medicine, 23:1921-1986.

10. 2. Handling of missing, unused or inconsistent data

In the analyses, for patients who are prematurely withdrawn, the last observation carried forward (LOCF) method will be used: This means that the results of the last post-baseline available assessment (Vend) will be analyzed (or that the baseline values will not be carried forward).

Missing data will be made available in the data management report.

The decision to replace missing items will be made prior to the end of the study.

10.3. General principles of outcome analysis

The analysis of data obtained from study subjects will be conducted by three main analysis approaches: intention to treat (ITT), full data analysis set (FAS) and per protocol (PP).

The ITT analysis includes all data obtained from subjects who have ever received the investigational drug at least once. This approach is used to analyze safety data related to adverse events.

The FAS analysis set is a group of subjects who have had the primary efficacy endpoint measured at least once since administration of the investigational drug, and all the measurement data is included in the analysis. However, the analysis excludes patients who have failed to meet the inclusion criteria or have taken any of prohibited concomitant drugs. In case missing data occurs at any point of time or if there are any subjects who drop out before the end of the study, data analysis is conducted for conservative assessment as if the latest data were obtained at such point of time (last observation carried forward).

The PP analysis includes data obtained from patients treated in compliance with the study protocol among those subjects included in the ITT analysis.

Efficacy data are analyzed primarily by the FAS approach and secondarily by the PP approach. The analysis of adverse event data is performed on an ITT basis, and laboratory test data are analyzed using an FAS (data itself) approach. The findings of those analyses are compared, but if any significant differences are found, the results of each analysis should be described.

10. 4. Statistical analysis methods

10.4.1. Demographics and medical history

Data from all the subjects who participate in this clinical study will be evaluated on a group-by-group basis. We will calculate the mean, standard deviation (SD), maximum and minimum values for continuous data and the absolute and relative frequencies for categorical data. Demographics and baseline data will be compared between two groups. Continuous variables are compared between groups and study centers by the t-test, Mann-Whitney test or ANOVA test and Kruskal-Wallis test, while categorical variables are compared by the Chi-square test and using the Mantel-Haenszel test at each center as a stratification factor. If any significant difference between two groups is accepted, the efficacy analysis will use the difference as a correct factor to correct heterogeneity between the groups.

10.4.2. Primary efficacy assessment

A t-test will be used to compare changes in frequency of urination (from baseline to the end of the study) between study groups on the voiding diary (see Annex 1) and calculate a 95% confidence interval for the difference between the groups. Further analysis, if necessary, will be conducted with a generalized linear model in order to correct heterogeneity between the groups.

10.4.2. Secondary efficacy assessment

Secondary efficacy endpoints (i.e., PPBC, OABSS and BSW) will be analyzed using the the Wilcoxon signed-rank sum test or paired t-test for continuous data and the McNemar test for categorical data. If necessary, a mixed effect model or generalized estimation equation will be used as an analysis tool to test the difference between study groups.

# **11. Ethics**

11. 1. Texts in force

The trial will be conducted in accordance with:

- The Declaration of Helsinki adopted by the World Medical Assembly in June 1964 and amended in Tokyo in October 1975, Venice in October 1983, Hong Kong in September 1989 and Somerset West in October 1996 (See Appendix 3); and
- ICH guidelines for Good Clinical Practice

11. 2. Patient information and consent

Before a patient is included in the trial, the objectives and methods of the trial are explained to him/her. The patient gives his/her consent in writing on given information.

11. 3. Submission to an Ethics Committee

The investigator/principal investigator will submit the documents required by the regulations according to his/her local ethics committee and will seek their advice in writing. Patients cannot be included until the approval of the ethics committee has been obtained.

If there are any amendments or modifications to the study protocol during the study, they should be known to the ethics committee. It should also be informed of any event likely to affect the safety of patients or compromise the continuation of the study.

11. 4. Confidentiality of the data

The patient’s right to confidentiality is maintained during data collection and processing.

# **12. References**

1. Abrams P, Cardozo L, Fall M, Griffiths D, Rosier P, Ulmsten U, van Kerrebroeck P, Victor A, Wein A. The standardization of terminology in lower urinary tract function: report from the standardization sub-committee of the International Continence Society. Neurourol Urodyn 2002;21:167-78

2. Irwin DE, Milsom I, Hunskaar S, Reilly K, Kopp Z, Herschorn S, Coyne K, Kelleher C, Hampel C, Artibani W, Abrams P. Population-based survey of urinary incontinence, overactive bladder, and other lower urinary tract symptoms in five countries: results of the EPIC study. Eur Urol 2006;50:1306-15

3. Einhoff V, Bavenden T, Glasser DB, Carlsson M, Eyland N, Roberts R. Symptom-specific efficacy of tolterodine extended release in patients with overactive bladder: the IMPACT trial. Int J Cli Pract 2006;60:745-51

4. Choo MS, Doo CK, Lee KS. Satisfaction with tolterodine: assessing symptom specific patient-reported goal achievement in the treatment of overactive bladder in female patients. (STARGATE study) 2008;62:191-6
